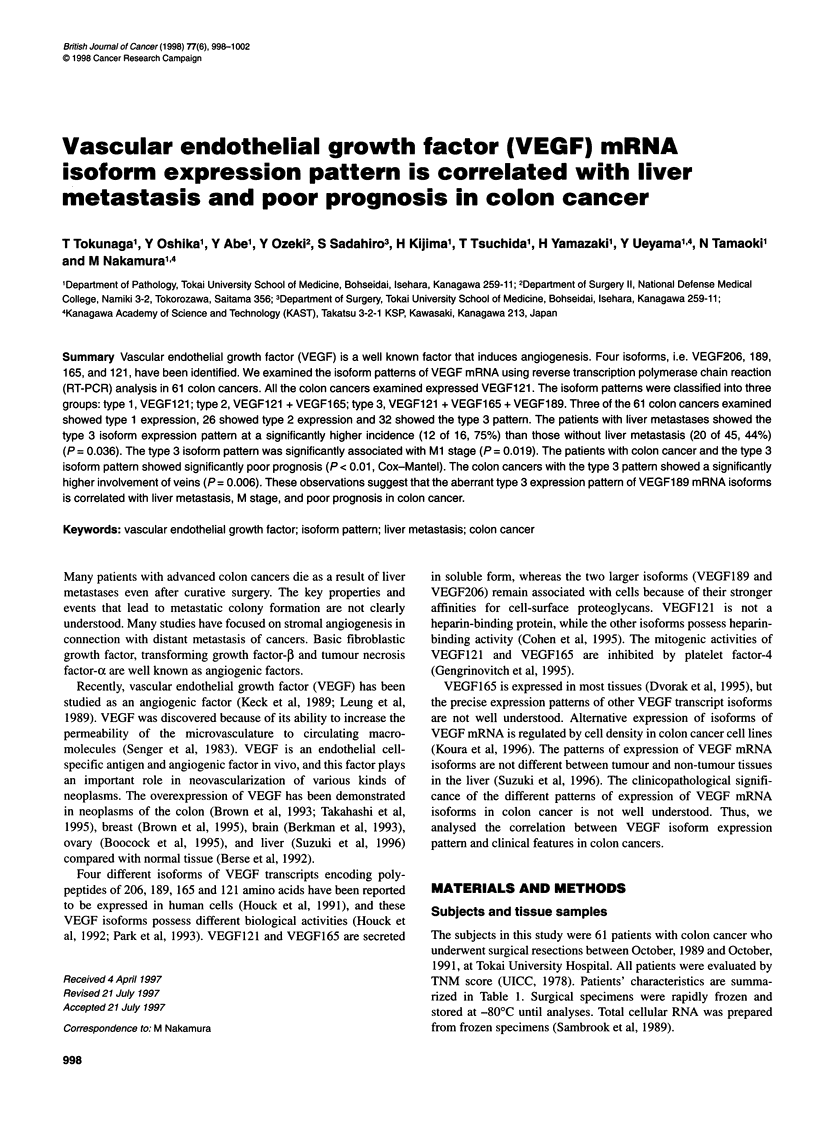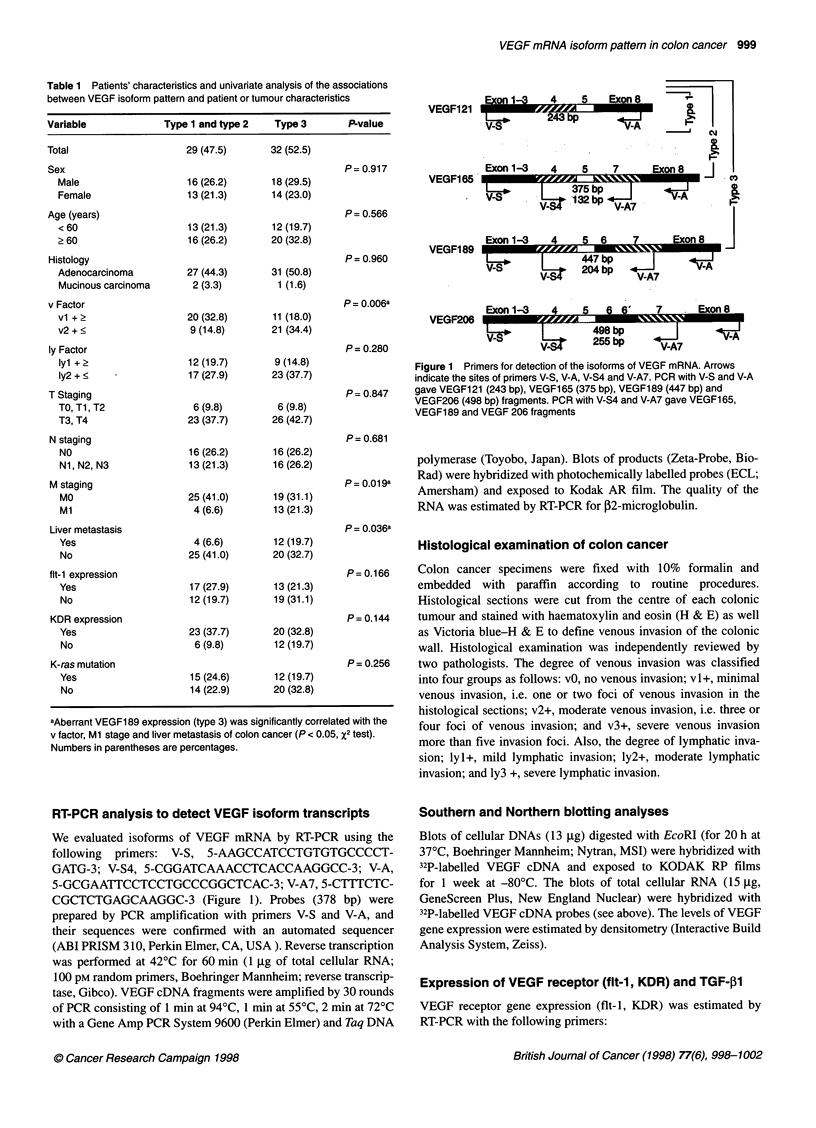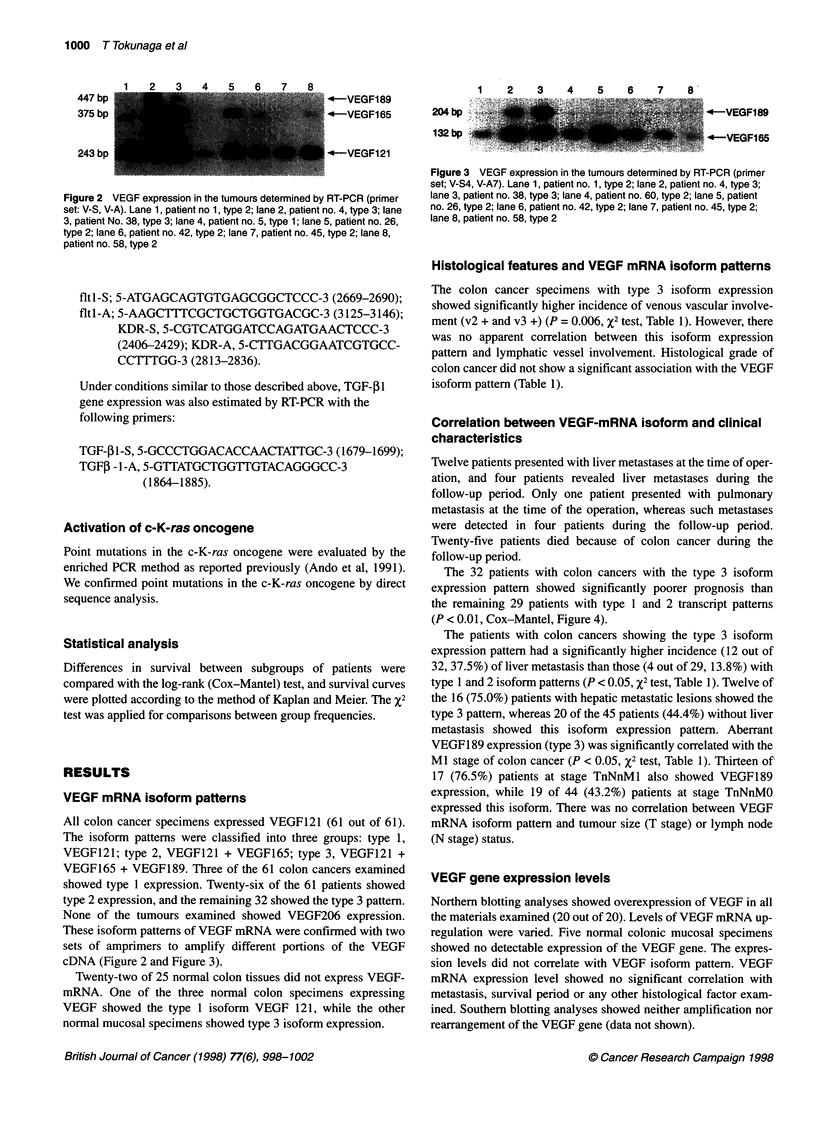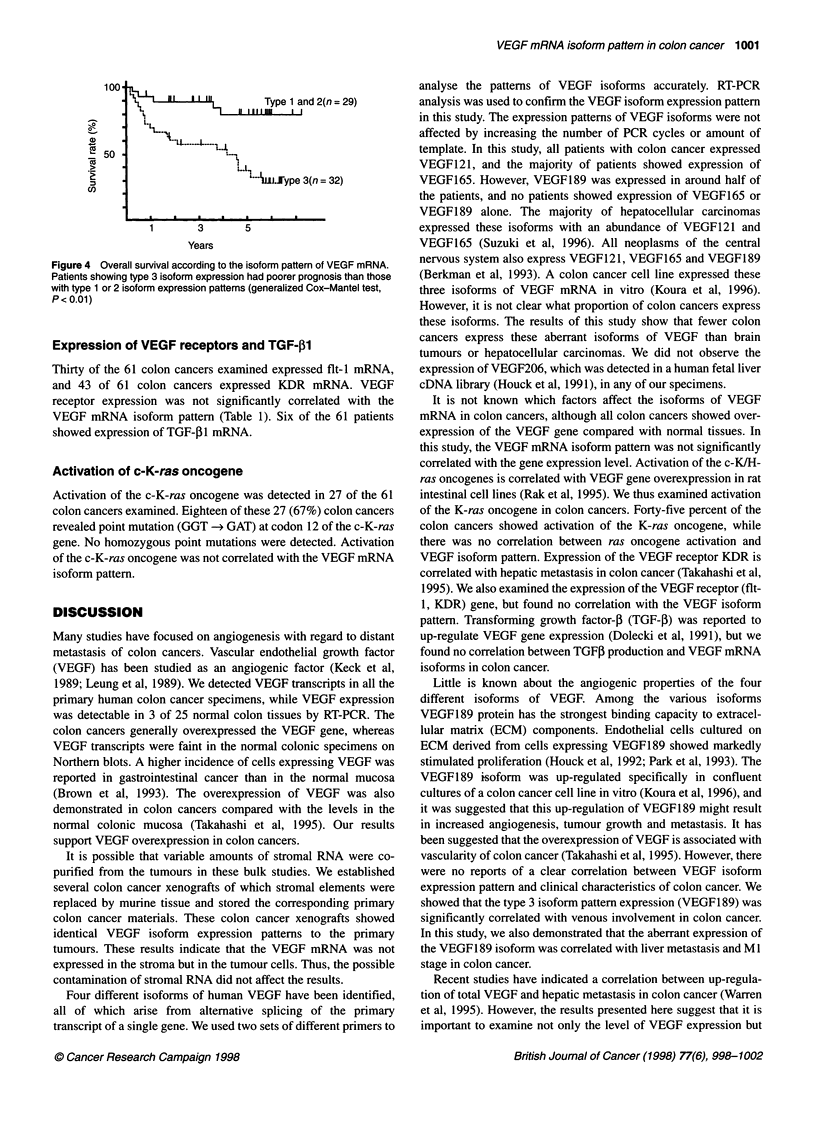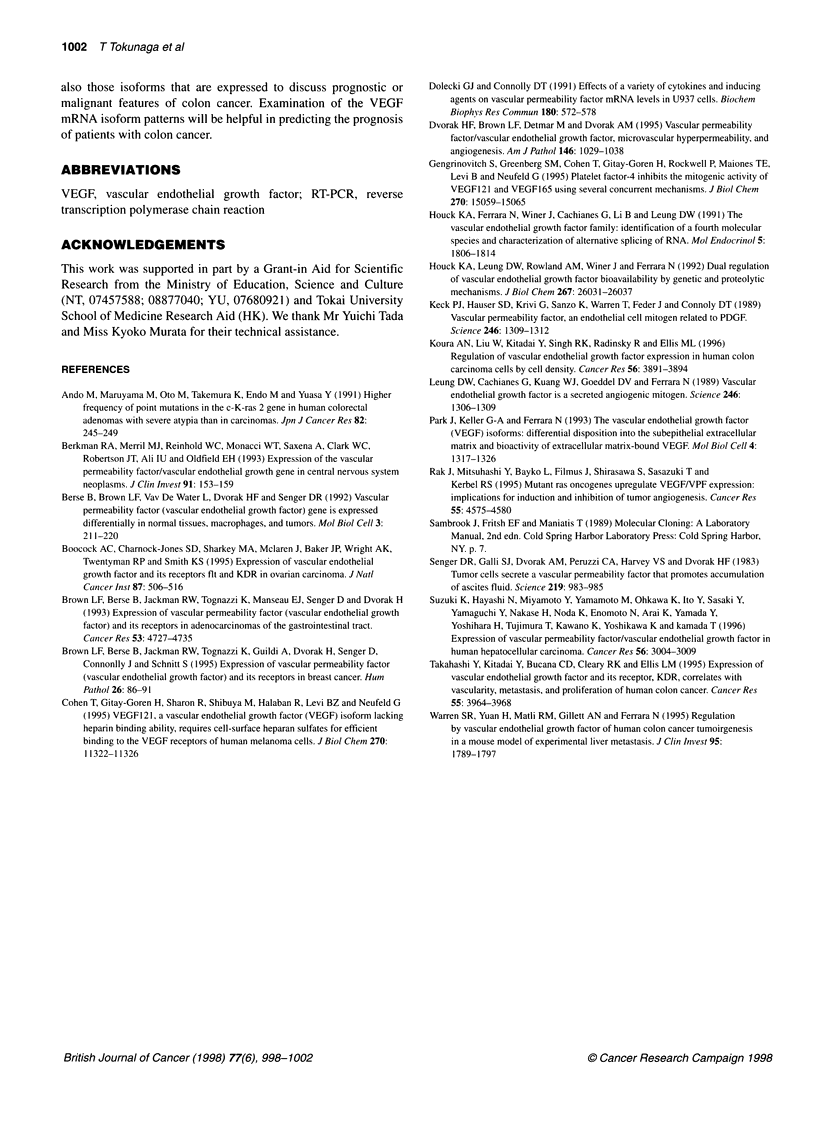# Vascular endothelial growth factor (VEGF) mRNA isoform expression pattern is correlated with liver metastasis and poor prognosis in colon cancer.

**DOI:** 10.1038/bjc.1998.164

**Published:** 1998-03

**Authors:** T. Tokunaga, Y. Oshika, Y. Abe, Y. Ozeki, S. Sadahiro, H. Kijima, T. Tsuchida, H. Yamazaki, Y. Ueyama, N. Tamaoki, M. Nakamura

**Affiliations:** Department of Pathology, Tokai University School of Medicine, Isehara, Kanagawa, Japan.

## Abstract

**Images:**


British Journal of Cancer (1998) 77(6), 998–1002
© 1998 Cancer Research Campaign

### Vascular endothelial growth factor (VEGF) mRNA isoform expression pattern is correlated with liver metastasis and poor prognosis in colon cancer

**T Tokunaga[1], Y Oshika[1], Y Abe[1], Y Ozeki[2], S Sadahiro[3], H Kijima[1], T Tsuchida[1], H Yamazaki[1], Y Ueyama[1,4], N Tamaoki[1] and M Nakamura[1,4]**

[1]Department of Pathology, Tokai University School of Medicine, Bohseidai, Isehara, Kanagawa 259-11; [2]Department of Surgery II, National Defense Medical College, Namiki 3-2, Tokorozawa, Saitama 356; [3]Department of Surgery, Tokai University School of Medicine, Bohseidai, Isehara, Kanagawa 259-11; [4]Kanagawa Academy of Science and Technology (KAST), Takatsu 3-2-1 KSP, Kawasaki, Kanagawa 213, Japan

**Summary** Vascular endothelial growth factor (VEGF) is a well known factor that induces angiogenesis. Four isoforms, i.e. VEGF206, 189, 165, and 121, have been identified. We examined the isoform patterns of VEGF mRNA using reverse transcription polymerase chain reaction (RT-PCR) analysis in 61 colon cancers. All the colon cancers examined expressed VEGF121. The isoform patterns were classified into three groups: type 1, VEGF121; type 2, VEGF121 + VEGF165; type 3, VEGF121 + VEGF165 + VEGF189. Three of the 61 colon cancers examined showed type 1 expression, 26 showed type 2 expression and 32 showed the type 3 pattern. The patients with liver metastases showed the type 3 isoform expression pattern at a significantly higher incidence (12 of 16, 75%) than those without liver metastasis (20 of 45, 44%) ($P = 0.036$). The type 3 isoform pattern was significantly associated with M1 stage ($P = 0.019$). The patients with colon cancer and the type 3 isoform pattern showed significantly poor prognosis ($P < 0.01$, Cox–Mantel). The colon cancers with the type 3 pattern showed a significantly higher involvement of veins ($P = 0.006$). These observations suggest that the aberrant type 3 expression pattern of VEGF189 mRNA isoforms is correlated with liver metastasis, M stage, and poor prognosis in colon cancer.

**Keywords:** vascular endothelial growth factor; isoform pattern; liver metastasis; colon cancer

Many patients with advanced colon cancers die as a result of liver metastases even after curative surgery. The key properties and events that lead to metastatic colony formation are not clearly understood. Many studies have focused on stromal angiogenesis in connection with distant metastasis of cancers. Basic fibroblastic growth factor, transforming growth factor-β and tumour necrosis factor-α are well known as angiogenic factors.

Recently, vascular endothelial growth factor (VEGF) has been studied as an angiogenic factor (Keck et al, 1989; Leung et al, 1989). VEGF was discovered because of its ability to increase the permeability of the microvasculature to circulating macromolecules (Senger et al, 1983). VEGF is an endothelial cell-specific antigen and angiogenic factor in vivo, and this factor plays an important role in neovascularization of various kinds of neoplasms. The overexpression of VEGF has been demonstrated in neoplasms of the colon (Brown et al, 1993; Takahashi et al, 1995), breast (Brown et al, 1995), brain (Berkman et al, 1993), ovary (Boocock et al, 1995), and liver (Suzuki et al, 1996) compared with normal tissue (Berse et al, 1992).

Four different isoforms of VEGF transcripts encoding polypeptides of 206, 189, 165 and 121 amino acids have been reported to be expressed in human cells (Houck et al, 1991), and these VEGF isoforms possess different biological activities (Houck et al, 1992; Park et al, 1993). VEGF121 and VEGF165 are secreted in soluble form, whereas the two larger isoforms (VEGF189 and VEGF206) remain associated with cells because of their stronger affinities for cell-surface proteoglycans. VEGF121 is not a heparin-binding protein, while the other isoforms possess heparin-binding activity (Cohen et al, 1995). The mitogenic activities of VEGF121 and VEGF165 are inhibited by platelet factor-4 (Gengrinovitch et al, 1995).

VEGF165 is expressed in most tissues (Dvorak et al, 1995), but the precise expression patterns of other VEGF transcript isoforms are not well understood. Alternative expression of isoforms of VEGF mRNA is regulated by cell density in colon cancer cell lines (Koura et al, 1996). The patterns of expression of VEGF mRNA isoforms are not different between tumour and non-tumour tissues in the liver (Suzuki et al, 1996). The clinicopathological significance of the different patterns of expression of VEGF mRNA isoforms in colon cancer is not well understood. Thus, we analysed the correlation between VEGF isoform expression pattern and clinical features in colon cancers.

### MATERIALS AND METHODS

### Subjects and tissue samples

The subjects in this study were 61 patients with colon cancer who underwent surgical resections between October, 1989 and October, 1991, at Tokai University Hospital. All patients were evaluated by TNM score (UICC, 1978). Patients' characteristics are summarized in Table 1. Surgical specimens were rapidly frozen and stored at –80°C until analyses. Total cellular RNA was prepared from frozen specimens (Sambrook et al, 1989).

Received 4 April 1997
Revised 21 July 1997
Accepted 21 July 1997

Correspondence to: M Nakamura

**Table 1** Patients' characteristics and univariate analysis of the associations between VEGF isoform pattern and patient or tumour characteristics

| Variable | Type 1 and type 2 | Type 3 | *P*-value |
|---|---|---|---|
| Total | 29 (47.5) | 32 (52.5) | |
| Sex | | | $P = 0.917$ |
| Male | 16 (26.2) | 18 (29.5) | |
| Female | 13 (21.3) | 14 (23.0) | |
| Age (years) | | | $P = 0.566$ |
| < 60 | 13 (21.3) | 12 (19.7) | |
| ≥ 60 | 16 (26.2) | 20 (32.8) | |
| Histology | | | $P = 0.960$ |
| Adenocarcinoma | 27 (44.3) | 31 (50.8) | |
| Mucinous carcinoma | 2 (3.3) | 1 (1.6) | |
| v Factor | | | $P = 0.006$[a] |
| v1 + ≥ | 20 (32.8) | 11 (18.0) | |
| v2 + ≤ | 9 (14.8) | 21 (34.4) | |
| ly Factor | | | $P = 0.280$ |
| ly1 + ≥ | 12 (19.7) | 9 (14.8) | |
| ly2 + ≤ | 17 (27.9) | 23 (37.7) | |
| T Staging | | | $P = 0.847$ |
| T0, T1, T2 | 6 (9.8) | 6 (9.8) | |
| T3, T4 | 23 (37.7) | 26 (42.7) | |
| N staging | | | $P = 0.681$ |
| N0 | 16 (26.2) | 16 (26.2) | |
| N1, N2, N3 | 13 (21.3) | 16 (26.2) | |
| M staging | | | $P = 0.019$[a] |
| M0 | 25 (41.0) | 19 (31.1) | |
| M1 | 4 (6.6) | 13 (21.3) | |
| Liver metastasis | | | $P = 0.036$[a] |
| Yes | 4 (6.6) | 12 (19.7) | |
| No | 25 (41.0) | 20 (32.7) | |
| flt-1 expression | | | $P = 0.166$ |
| Yes | 17 (27.9) | 13 (21.3) | |
| No | 12 (19.7) | 19 (31.1) | |
| KDR expression | | | $P = 0.144$ |
| Yes | 23 (37.7) | 20 (32.8) | |
| No | 6 (9.8) | 12 (19.7) | |
| K-*ras* mutation | | | $P = 0.256$ |
| Yes | 15 (24.6) | 12 (19.7) | |
| No | 14 (22.9) | 20 (32.8) | |

[a]Aberrant VEGF189 expression (type 3) was significantly correlated with the v factor, M1 stage and liver metastasis of colon cancer ($P < 0.05$, $\chi^2$ test). Numbers in parentheses are percentages.

### RT-PCR analysis to detect VEGF isoform transcripts

We evaluated isoforms of VEGF mRNA by RT-PCR using the following primers: V-S, 5-AAGCCATCCTGTGTGCCCCT-GATG-3; V-S4, 5-CGGATCAAACCTCACCAAGGCC-3; V-A, 5-GCGAATTCCTCCTGCCCGGCTCAC-3; V-A7, 5-CTTTCTC-CGCTCTGAGCAAGGC-3 (Figure 1). Probes (378 bp) were prepared by PCR amplification with primers V-S and V-A, and their sequences were confirmed with an automated sequencer (ABI PRISM 310, Perkin Elmer, CA, USA ). Reverse transcription was performed at 42°C for 60 min (1 μg of total cellular RNA; 100 pM random primers, Boehringer Mannheim; reverse transcriptase, Gibco). VEGF cDNA fragments were amplified by 30 rounds of PCR consisting of 1 min at 94°C, 1 min at 55°C, 2 min at 72°C with a Gene Amp PCR System 9600 (Perkin Elmer) and *Taq* DNA polymerase (Toyobo, Japan). Blots of products (Zeta-Probe, Bio-Rad) were hybridized with photochemically labelled probes (ECL; Amersham) and exposed to Kodak AR film. The quality of the RNA was estimated by RT-PCR for β2-microglobulin.

**Figure 1** Primers for detection of the isoforms of VEGF mRNA. Arrows indicate the sites of primers V-S, V-A, V-S4 and V-A7. PCR with V-S and V-A gave VEGF121 (243 bp), VEGF165 (375 bp), VEGF189 (447 bp) and VEGF206 (498 bp) fragments. PCR with V-S4 and V-A7 gave VEGF165, VEGF189 and VEGF 206 fragments

### Histological examination of colon cancer

Colon cancer specimens were fixed with 10% formalin and embedded with paraffin according to routine procedures. Histological sections were cut from the centre of each colonic tumour and stained with haematoxylin and eosin (H & E) as well as Victoria blue–H & E to define venous invasion of the colonic wall. Histological examination was independently reviewed by two pathologists. The degree of venous invasion was classified into four groups as follows: v0, no venous invasion; v1+, minimal venous invasion, i.e. one or two foci of venous invasion in the histological sections; v2+, moderate venous invasion, i.e. three or four foci of venous invasion; and v3+, severe venous invasion more than five invasion foci. Also, the degree of lymphatic invasion; ly1+, mild lymphatic invasion; ly2+, moderate lymphatic invasion; and ly3 +, severe lymphatic invasion.

### Southern and Northern blotting analyses

Blots of cellular DNAs (13 μg) digested with *Eco*RI (for 20 h at 37°C, Boehringer Mannheim; Nytran, MSI) were hybridized with $^{32}P$-labelled VEGF cDNA and exposed to KODAK RP films for 1 week at –80°C. The blots of total cellular RNA (15 μg, GeneScreen Plus, New England Nuclear) were hybridized with $^{32}P$-labelled VEGF cDNA probes (see above). The levels of VEGF gene expression were estimated by densitometry (Interactive Build Analysis System, Zeiss).

### Expression of VEGF receptor (flt-1, KDR) and TGF-β1

VEGF receptor gene expression (flt-1, KDR) was estimated by RT-PCR with the following primers:

© Cancer Research Campaign 1998

**Figure 2** VEGF expression in the tumours determined by RT-PCR (primer set: V-S, V-A). Lane 1, patient no 1, type 2; lane 2, patient no. 4, type 3; lane 3, patient No. 38, type 3; lane 4, patient no. 5, type 1; lane 5, patient no. 26, type 2; lane 6, patient no. 42, type 2; lane 7, patient no. 45, type 2; lane 8, patient no. 58, type 2

flt1-S; 5-ATGAGCAGTGTGAGCGGCTCCC-3 (2669–2690);
flt1-A; 5-AAGCTTTCGCTGCTGGTGACGC-3 (3125–3146);
KDR-S, 5-CGTCATGGATCCAGATGAACTCCC-3 (2406–2429); KDR-A, 5-CTTGACGGAATCGTGCCCCTTTGG-3 (2813–2836).

Under conditions similar to those described above, TGF-β1 gene expression was also estimated by RT-PCR with the following primers:

TGF-β1-S, 5-GCCCTGGACACCAACTATTGC-3 (1679–1699);
TGFβ -1-A, 5-GTTATGCTGGTTGTACAGGGCC-3 (1864–1885).

### Activation of c-K-*ras* oncogene

Point mutations in the c-K-*ras* oncogene were evaluated by the enriched PCR method as reported previously (Ando et al, 1991). We confirmed point mutations in the c-K-*ras* oncogene by direct sequence analysis.

### Statistical analysis

Differences in survival between subgroups of patients were compared with the log-rank (Cox–Mantel) test, and survival curves were plotted according to the method of Kaplan and Meier. The $\chi^2$ test was applied for comparisons between group frequencies.

### RESULTS

### VEGF mRNA isoform patterns

All colon cancer specimens expressed VEGF121 (61 out of 61). The isoform patterns were classified into three groups: type 1, VEGF121; type 2, VEGF121 + VEGF165; type 3, VEGF121 + VEGF165 + VEGF189. Three of the 61 colon cancers examined showed type 1 expression. Twenty-six of the 61 patients showed type 2 expression, and the remaining 32 showed the type 3 pattern. None of the tumours examined showed VEGF206 expression. These isoform patterns of VEGF mRNA were confirmed with two sets of amprimers to amplify different portions of the VEGF cDNA (Figure 2 and Figure 3).

Twenty-two of 25 normal colon tissues did not express VEGF-mRNA. One of the three normal colon specimens expressing VEGF showed the type 1 isoform VEGF 121, while the other normal mucosal specimens showed type 3 isoform expression.

**Figure 3** VEGF expression in the tumours determined by RT-PCR (primer set; V-S4, V-A7). Lane 1, patient no. 1, type 2; lane 2, patient no. 4, type 3; lane 3, patient no. 38, type 3; lane 4, patient no. 60, type 2; lane 5, patient no. 26, type 2; lane 6, patient no. 42, type 2; lane 7, patient no. 45, type 2; lane 8, patient no. 58, type 2

### Histological features and VEGF mRNA isoform patterns

The colon cancer specimens with type 3 isoform expression showed significantly higher incidence of venous vascular involvement (v2 + and v3 +) ($P = 0.006$, $\chi^2$ test, Table 1). However, there was no apparent correlation between this isoform expression pattern and lymphatic vessel involvement. Histological grade of colon cancer did not show a significant association with the VEGF isoform pattern (Table 1).

### Correlation between VEGF-mRNA isoform and clinical characteristics

Twelve patients presented with liver metastases at the time of operation, and four patients revealed liver metastases during the follow-up period. Only one patient presented with pulmonary metastasis at the time of the operation, whereas such metastases were detected in four patients during the follow-up period. Twenty-five patients died because of colon cancer during the follow-up period.

The 32 patients with colon cancers with the type 3 isoform expression pattern showed significantly poorer prognosis than the remaining 29 patients with type 1 and 2 transcript patterns ($P < 0.01$, Cox–Mantel, Figure 4).

The patients with colon cancers showing the type 3 isoform expression pattern had a significantly higher incidence (12 out of 32, 37.5%) of liver metastasis than those (4 out of 29, 13.8%) with type 1 and 2 isoform patterns ($P < 0.05$, $\chi^2$ test, Table 1). Twelve of the 16 (75.0%) patients with hepatic metastatic lesions showed the type 3 pattern, whereas 20 of the 45 patients (44.4%) without liver metastasis showed this isoform expression pattern. Aberrant VEGF189 expression (type 3) was significantly correlated with the M1 stage of colon cancer ($P < 0.05$, $\chi^2$ test, Table 1). Thirteen of 17 (76.5%) patients at stage TnNnM1 also showed VEGF189 expression, while 19 of 44 (43.2%) patients at stage TnNnM0 expressed this isoform. There was no correlation between VEGF mRNA isoform pattern and tumour size (T stage) or lymph node (N stage) status.

### VEGF gene expression levels

Northern blotting analyses showed overexpression of VEGF in all the materials examined (20 out of 20). Levels of VEGF mRNA up-regulation were varied. Five normal colonic mucosal specimens showed no detectable expression of the VEGF gene. The expression levels did not correlate with VEGF isoform pattern. VEGF mRNA expression level showed no significant correlation with metastasis, survival period or any other histological factor examined. Southern blotting analyses showed neither amplification nor rearrangement of the VEGF gene (data not shown).

© Cancer Research Campaign 1998

**Figure 4** Overall survival according to the isoform pattern of VEGF mRNA. Patients showing type 3 isoform expression had poorer prognosis than those with type 1 or 2 isoform expression patterns (generalized Cox–Mantel test, $P < 0.01$)

### Expression of VEGF receptors and TGF-β1

Thirty of the 61 colon cancers examined expressed flt-1 mRNA, and 43 of 61 colon cancers expressed KDR mRNA. VEGF receptor expression was not significantly correlated with the VEGF mRNA isoform pattern (Table 1). Six of the 61 patients showed expression of TGF-β1 mRNA.

### Activation of c-K-*ras* oncogene

Activation of the c-K-*ras* oncogene was detected in 27 of the 61 colon cancers examined. Eighteen of these 27 (67%) colon cancers revealed point mutation (GGT → GAT) at codon 12 of the c-K-*ras* gene. No homozygous point mutations were detected. Activation of the c-K-*ras* oncogene was not correlated with the VEGF mRNA isoform pattern.

### DISCUSSION

Many studies have focused on angiogenesis with regard to distant metastasis of colon cancers. Vascular endothelial growth factor (VEGF) has been studied as an angiogenic factor (Keck et al, 1989; Leung et al, 1989). We detected VEGF transcripts in all the primary human colon cancer specimens, while VEGF expression was detectable in 3 of 25 normal colon tissues by RT-PCR. The colon cancers generally overexpressed the VEGF gene, whereas VEGF transcripts were faint in the normal colonic specimens on Northern blots. A higher incidence of cells expressing VEGF was reported in gastrointestinal cancer than in the normal mucosa (Brown et al, 1993). The overexpression of VEGF was also demonstrated in colon cancers compared with the levels in the normal colonic mucosa (Takahashi et al, 1995). Our results support VEGF overexpression in colon cancers.

It is possible that variable amounts of stromal RNA were co-purified from the tumours in these bulk studies. We established several colon cancer xenografts of which stromal elements were replaced by murine tissue and stored the corresponding primary colon cancer materials. These colon cancer xenografts showed identical VEGF isoform expression patterns to the primary tumours. These results indicate that the VEGF mRNA was not expressed in the stroma but in the tumour cells. Thus, the possible contamination of stromal RNA did not affect the results.

Four different isoforms of human VEGF have been identified, all of which arise from alternative splicing of the primary transcript of a single gene. We used two sets of different primers to analyse the patterns of VEGF isoforms accurately. RT-PCR analysis was used to confirm the VEGF isoform expression pattern in this study. The expression patterns of VEGF isoforms were not affected by increasing the number of PCR cycles or amount of template. In this study, all patients with colon cancer expressed VEGF121, and the majority of patients showed expression of VEGF165. However, VEGF189 was expressed in around half of the patients, and no patients showed expression of VEGF165 or VEGF189 alone. The majority of hepatocellular carcinomas expressed these isoforms with an abundance of VEGF121 and VEGF165 (Suzuki et al, 1996). All neoplasms of the central nervous system also express VEGF121, VEGF165 and VEGF189 (Berkman et al, 1993). A colon cancer cell line expressed these three isoforms of VEGF mRNA in vitro (Koura et al, 1996). However, it is not clear what proportion of colon cancers express these isoforms. The results of this study show that fewer colon cancers express these aberrant isoforms of VEGF than brain tumours or hepatocellular carcinomas. We did not observe the expression of VEGF206, which was detected in a human fetal liver cDNA library (Houck et al, 1991), in any of our specimens.

It is not known which factors affect the isoforms of VEGF mRNA in colon cancers, although all colon cancers showed over-expression of the VEGF gene compared with normal tissues. In this study, the VEGF mRNA isoform pattern was not significantly correlated with the gene expression level. Activation of the c-K/H-*ras* oncogenes is correlated with VEGF gene overexpression in rat intestinal cell lines (Rak et al, 1995). We thus examined activation of the K-*ras* oncogene in colon cancers. Forty-five percent of the colon cancers showed activation of the K-*ras* oncogene, while there was no correlation between *ras* oncogene activation and VEGF isoform pattern. Expression of the VEGF receptor KDR is correlated with hepatic metastasis in colon cancer (Takahashi et al, 1995). We also examined the expression of the VEGF receptor (flt-1, KDR) gene, but found no correlation with the VEGF isoform pattern. Transforming growth factor-β (TGF-β) was reported to up-regulate VEGF gene expression (Dolecki et al, 1991), but we found no correlation between TGFβ production and VEGF mRNA isoforms in colon cancer.

Little is known about the angiogenic properties of the four different isoforms of VEGF. Among the various isoforms VEGF189 protein has the strongest binding capacity to extracellular matrix (ECM) components. Endothelial cells cultured on ECM derived from cells expressing VEGF189 showed markedly stimulated proliferation (Houck et al, 1992; Park et al, 1993). The VEGF189 isoform was up-regulated specifically in confluent cultures of a colon cancer cell line in vitro (Koura et al, 1996), and it was suggested that this up-regulation of VEGF189 might result in increased angiogenesis, tumour growth and metastasis. It has been suggested that the overexpression of VEGF is associated with vascularity of colon cancer (Takahashi et al, 1995). However, there were no reports of a clear correlation between VEGF isoform expression pattern and clinical characteristics of colon cancer. We showed that the type 3 isoform pattern expression (VEGF189) was significantly correlated with venous involvement in colon cancer. In this study, we also demonstrated that the aberrant expression of the VEGF189 isoform was correlated with liver metastasis and M1 stage in colon cancer.

Recent studies have indicated a correlation between up-regulation of total VEGF and hepatic metastasis in colon cancer (Warren et al, 1995). However, the results presented here suggest that it is important to examine not only the level of VEGF expression but also those isoforms that are expressed to discuss prognostic or malignant features of colon cancer. Examination of the VEGF mRNA isoform patterns will be helpful in predicting the prognosis of patients with colon cancer.

### ABBREVIATIONS

VEGF, vascular endothelial growth factor; RT-PCR, reverse transcription polymerase chain reaction

### ACKNOWLEDGEMENTS

This work was supported in part by a Grant-in Aid for Scientific Research from the Ministry of Education, Science and Culture (NT, 07457588; 08877040; YU, 07680921) and Tokai University School of Medicine Research Aid (HK). We thank Mr Yuichi Tada and Miss Kyoko Murata for their technical assistance.

### REFERENCES

Ando M, Maruyama M, Oto M, Takemura K, Endo M and Yuasa Y (1991) Higher frequency of point mutations in the c-K-ras 2 gene in human colorectal adenomas with severe atypia than in carcinomas. *Jpn J Cancer Res* **82**: 245–249

Berkman RA, Merril MJ, Reinhold WC, Monacci WT, Saxena A, Clark WC, Robertson JT, Ali IU and Oldfield EH (1993) Expression of the vascular permeability factor/vascular endothelial growth gene in central nervous system neoplasms. *J Clin Invest* **91**: 153–159

Berse B, Brown LF, Vav De Water L, Dvorak HF and Senger DR (1992) Vascular permeability factor (vascular endothelial growth factor) gene is expressed differentially in normal tissues, macrophages, and tumors. *Mol Biol Cell* **3**: 211–220

Boocock AC, Charnock-Jones SD, Sharkey MA, Mclaren J, Baker JP, Wright AK, Twentyman RP and Smith KS (1995) Expression of vascular endothelial growth factor and its receptors flt and KDR in ovarian carcinoma. *J Natl Cancer Inst* **87**: 506–516

Brown LF, Berse B, Jackman RW, Tognazzi K, Manseau EJ, Senger D and Dvorak H (1993) Expression of vascular permeability factor (vascular endothelial growth factor) and its receptors in adenocarcinomas of the gastrointestinal tract. *Cancer Res* **53**: 4727–4735

Brown LF, Berse B, Jackman RW, Tognazzi K, Guildi A, Dvorak H, Senger D, Connonlly J and Schnitt S (1995) Expression of vascular permeability factor (vascular endothelial growth factor) and its receptors in breast cancer. *Hum Pathol* **26**: 86–91

Cohen T, Gitay-Goren H, Sharon R, Shibuya M, Halaban R, Levi BZ and Neufeld G (1995) VEGF121, a vascular endothelial growth factor (VEGF) isoform lacking heparin binding ability, requires cell-surface heparan sulfates for efficient binding to the VEGF receptors of human melanoma cells. *J Biol Chem* **270**: 11322–11326

Dolecki GJ and Connolly DT (1991) Effects of a variety of cytokines and inducing agents on vascular permeability factor mRNA levels in U937 cells. *Biochem Biophys Res Commun* **180**: 572–578

Dvorak HF, Brown LF, Detmar M and Dvorak AM (1995) Vascular permeability factor/vascular endothelial growth factor, microvascular hyperpermeability, and angiogenesis. *Am J Pathol* **146**: 1029–1038

Gengrinovitch S, Greenberg SM, Cohen T, Gitay-Goren H, Rockwell P, Maiones TE, Levi B and Neufeld G (1995) Platelet factor-4 inhibits the mitogenic activity of VEGF121 and VEGF165 using several concurrent mechanisms. *J Biol Chem* **270**: 15059–15065

Houck KA, Ferrara N, Winer J, Cachianes G, Li B and Leung DW (1991) The vascular endothelial growth factor family: identification of a fourth molecular species and characterization of alternative splicing of RNA. *Mol Endocrinol* **5**: 1806–1814

Houck KA, Leung DW, Rowland AM, Winer J and Ferrara N (1992) Dual regulation of vascular endothelial growth factor bioavailability by genetic and proteolytic mechanisms. *J Biol Chem* **267**: 26031–26037

Keck PJ, Hauser SD, Krivi G, Sanzo K, Warren T, Feder J and Connoly DT (1989) Vascular permeability factor, an endothelial cell mitogen related to PDGF. *Science* **246**: 1309–1312

Koura AN, Liu W, Kitadai Y, Singh RK, Radinsky R and Ellis ML (1996) Regulation of vascular endothelial growth factor expression in human colon carcinoma cells by cell density. *Cancer Res* **56**: 3891–3894

Leung DW, Cachianes G, Kuang WJ, Goeddel DV and Ferrara N (1989) Vascular endothelial growth factor is a secreted angiogenic mitogen. *Science* **246**: 1306–1309

Park J, Keller G-A and Ferrara N (1993) The vascular endothelial growth factor (VEGF) isoforms: differential disposition into the subepithelial extracellular matrix and bioactivity of extracellular matrix-bound VEGF. *Mol Biol Cell* **4**: 1317–1326

Rak J, Mitsuhashi Y, Bayko L, Filmus J, Shirasawa S, Sasazuki T and Kerbel RS (1995) Mutant ras oncogenes upregulate VEGF/VPF expression: implications for induction and inhibition of tumor angiogenesis. *Cancer Res* **55**: 4575–4580

Sambrook J, Fritsh EF and Maniatis T (1989) Molecular Cloning: A Laboratory Manual, 2nd edn. Cold Spring Harbor Laboratory Press: Cold Spring Harbor, NY. p. 7.

Senger DR, Galli SJ, Dvorak AM, Peruzzi CA, Harvey VS and Dvorak HF (1983) Tumor cells secrete a vascular permeability factor that promotes accumulation of ascites fluid. *Science* **219**: 983–985

Suzuki K, Hayashi N, Miyamoto Y, Yamamoto M, Ohkawa K, Ito Y, Sasaki Y, Yamaguchi Y, Nakase H, Noda K, Enomoto N, Arai K, Yamada Y, Yoshihara H, Tujimura T, Kawano K, Yoshikawa K and kamada T (1996) Expression of vascular permeability factor/vascular endothelial growth factor in human hepatocellular carcinoma. *Cancer Res* **56**: 3004–3009

Takahashi Y, Kitadai Y, Bucana CD, Cleary RK and Ellis LM (1995) Expression of vascular endothelial growth factor and its receptor, KDR, correlates with vascularity, metastasis, and proliferation of human colon cancer. *Cancer Res* **55**: 3964–3968

Warren SR, Yuan H, Matli RM, Gillett AN and Ferrara N (1995) Regulation by vascular endothelial growth factor of human colon cancer tumoirgenesis in a mouse model of experimental liver metastasis. *J Clin Invest* **95**: 1789–1797

© Cancer Research Campaign 1998